# Tritordeum as an Innovative Alternative to Wheat: A Comparative Digestion Study on Bread

**DOI:** 10.3390/molecules27041308

**Published:** 2022-02-15

**Authors:** Chiara Nitride, Giovanni D’Auria, Andrea Dente, Viola Landolfi, Gianluca Picariello, Gianfranco Mamone, Massimo Blandino, Raffaele Romano, Pasquale Ferranti

**Affiliations:** 1 Department of Agricultural Sciences, University of Naples Federico II, Via Università 100, 80055 Portici, Italy; chiara.nitride@unina.it (C.N.); and.dente@studenti.unina.it (A.D.); raffaele.romano@unina.it (R.R.); ferranti@unina.it (P.F.); 2 Department of Agricultural, Forest and Food Sciences, University of Turin, Largo Braccini 2, 10095 Grugliasco, Italy; viola.landolfi@unito.it (V.L.); massimo.blandino@unito.it (M.B.); 3 Institute of Food Science, National Research Council, 83100 Avellino, Italy; picariello@isa.cnr.it (G.P.); mamone@isa.cnr.it (G.M.)

**Keywords:** tritordeum, in vitro digestion, peptidomics, alpha amino nitrogen, R5, wheat allergy, celiac disease

## Abstract

Tritordeum results from the crossbreeding of a wild barley (*Hordeum chilense*) species with durum wheat (*Triticum turgidum* spp. *turgidum*). This hexaploid crop exhibits agronomic and rheological characteristics like soft wheat, resulting in an innovative raw material to produce baked goods. We applied a gel-based proteomic approach on refined flours to evaluate protein expression differences among two widespread tritordeum cultivars (Aucan and Bulel) taking as the reference semolina and flour derived from a durum and a soft wheat cvs, respectively. The products of in vitro digestion of model breads were analyzed to compare bio-accessibility of nutrients and mapping tritordeum bread resistant peptides. Significant differences among the protein profiles of the four flours were highlighted by electrophoresis. The amino acid bio-accessibility and the reducing sugars of tritordeum and wheat breads were comparable. Tritordeum cvs had about 15% higher alpha-amino nitrogen released at the end of the duodenal simulated digestion than soft wheat (*p* < 0.05). Bulel tritordeum flour, bread and digested bread had about 55% less R5-epitopes compared to the soft wheat. Differences in protein expression found between the two tritordeum cvs reflected in diverse digestion products and allergenic and celiacogenic potential of the duodenal peptides. Proteomic studies of a larger number of tritordeum cvs may be successful in selecting those with good agronomical performances and nutritional advantages.

## 1. Introduction

Wheat grains are the world’s most important staple food crop. The derived flour is a key ingredient in the preparation of bakery and pasta products, accounting for 20% of the total dietary calories and proteins in the human diet [1]. Throughout the centuries, the natural selection and hybridization among different wheat varieties, aimed at obtaining species easy to harvest and high in yield, have led to the modern tetraploid durum (*Triticum turgidum* spp. *durum*, AABB) and hexaploid bread wheat (*Triticum aestivum* spp. *aestivum*, AABBDD) [2]. The fast global changes of the last decade have made agricultural productivity more uncertain. Particularly, rising temperatures and decreased water availability are primary reasons for crop yield losses and reductions in the area harvested [3,4,5]. Barley is a crop that is adapted to a wide range of environmental landscapes, including high altitude and high latitude regions and to saline and dry conditions. Furthermore, barley flour has low machinability and bread-making performance compared to wheat. It was found that barley breads have an increased viscosity of the bolus, due to the presence of resistant starch and fibers, that reduced enzymes’ accessibility and therefore slowed the in vitro static and dynamic starch digestion [6]. Since the beginning of the twentieth century, cereal breeders have focused their efforts on the development of interspecific wheat hybrids to obtain new cereals with increased phytochemical content, improved agronomic performances and technological qualities. The hexaploid hybrid tritordeum (x *tritordeum martini*, AABBHchHch) is the product of cross-breeding *Hordeum chilense*, a South American wild barley species, and durum wheat. This hybridization aimed to combine the excellent traits of the *Hordeum*, such as high endosperm carotenoid content and higher tolerance to biotic and abiotic stress, with the technological qualities of wheat [7]. Tritordeum is today commercialized as an innovative alternative to conventional small cereal crops (www.tritordeum.com) [8,9], with rheological and baking performances similar to bread wheat [10]. Interestingly, in a clinical study involving subjects with non-celiac gluten sensitivity (NCGS), tritordeum breads were sensorially more appreciated than the gluten-free counterpart, showing good gastrointestinal tolerance [8,11]. 

A few recent studies in Europe have been focused on the agronomical traits, looking at yield performance of tritordeum cultivars (cvs) over conventional soft and durum wheats [7,12,13]. Scientific works have so far mainly focused on the bioactive compounds’ content: tritordeum, in fact, has higher levels of carotenoids and arabinoxylans than wheat, and these result in a greater total antioxidant activity. Tritordeum has twice the amount of β-glucans compared to durum wheat, although a similar amount to soft wheat, but significantly higher arabinoxylans [14] with prebiotic, immunomodulatory, antitumor, and anti-inflammatory activities [15,16]. Grain protein content (GPC) and gluten composition, in addition to the nutritional traits, play a major role in conferring the technological properties of wheat and other small cereals to the dough. By comparing the GPC of tritordeum with that of durum and soft wheat in different climatic conditions, tritordeum results in a higher GPC, ranging between 11% and 17%. Tritordeum produced by organic farming showed higher GPC than durum wheat with a larger number of high-molecular-weight glutenin subunits [13]. Although one of the parental lines of tritordeum is a durum wheat cultivar (cv), the similarity, in terms of derived flour quality, is much closer to that of hexaploid soft wheat, with specific interest for bread-making and baking processes [13].

Furthermore, little is known about the protein level differences across cvs or regarding the tritordeum protein digestibility. 

This study aims at comparing the protein profile of two tritordeum cvs, Bulel and Aucan, with soft wheat cv Altamira and a durum wheat cv, Antalis. Model breads were used to compare the digestion products (free-amino nitrogen and free glucose) using an in vitro digestion that included a standardized oral, gastric, and duodenal model simulating the physiological conditions of a healthy adult [17]. 

This work represents the first molecular characterization by advanced mass spectrometry of the peptides resistant to digestion of tritordeum bread, which was prepared with the two most common cvs, namely Aucan and Bulel. We mapped the contribution of the parent *H. chilense* to the tritordeum proteome and evaluated in silico the presence of peptides related to celiac toxicity and allergenicity. The immunoreactivity of the R5 monoclonal antibody targeting the celiacogenic sequence “QQPFP” was studied in the flours, as well as in undigested and digested bread.

## 2. Results and Discussion

### 2.1. Flour Protein Characterization

Flour from two tritordeum cvs, Aucan and Bulel, which are the most widely cultivated in Europe and agronomically characterized, were compared to flour obtained from two wheat cvs, Altamira (soft wheat) and Antalis (durum wheat), for mapping differences in protein content and quality.

The durum wheat, Antalis cv, and tritordeum, Aucan and Bulel cvs, had a total protein content (TPC) higher than the soft wheat, Altamira cv (*p* < 0.05) (Table 1). 

Aucan also had the highest ash amount. These data are in line with a previous study looking at the adaptability of tritordeum cvs in the east Mediterranean region, showing Aucan with a higher protein content compared to Bulel and to the soft wheat cv Falado [11]. 

**Figure 1:**
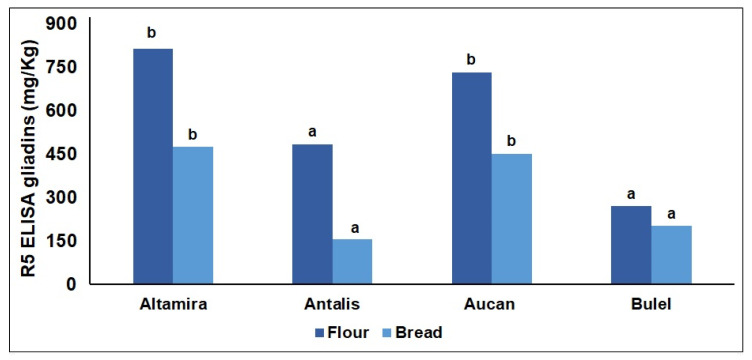
Quantification of the “QQPFP” celiac toxic motif recognized by the R5 monoclonal antibody of proteins extracted from the flours and the model bread obtained from different cereals. Within each product, flour and bread, bars with different letters are significantly different (*p* < 0.05) and the REGW-F test.

The concentration of gliadins in the flours was determined using a commercial sandwich ELISA test kit with the R5 monoclonal antibody to target the QQPFP celiac toxic motif (Figure 1) and the homologous LQPFP, QLPYP, QQSFP, QQTFP, PQPFPF, QQPYP, and PQPFP to a lower degree [18]. The toxic sequence appears repeatedly in the ω-, γ-, and α/β-gliadins [19]. The data are expressed as mg of R5 gliadin per kg of flour and the mg of gluten can be extrapolated using a conversion factor of two as suggested by the ELISA manufacturer. Despite the fact that the Altamira cv showed the lowest TPC, this soft wheat had the highest R5-gliadin concentration, which was comparable to Aucan, which had a significantly higher protein content. The durum wheat cv Antalis had a 40% lower content of R5-gliadin concentration per kg of flour compared to soft wheat cv Altamira. These results were in line with literature data showing the gluten content in tritordeum to be comparable or even higher to bread wheats, with Aucan higher in gluten compared with Bulel by four percentage points [11]. Interestingly, Bulel showed a 66% lower R5 immunoreactivity per kg of flour compared to Aucan. The reduced immunoreactivity of Bulel cv underlies important differences in terms of R5-gliadin sequences between the two tritordeum cvs. The analytical methodologies available for gluten determination in wheat may not be appropriate for all tritordeum cvs, since they may lead to an underestimation of the gluten content due to structural differences of the protein sequences.

**Figure 2:**
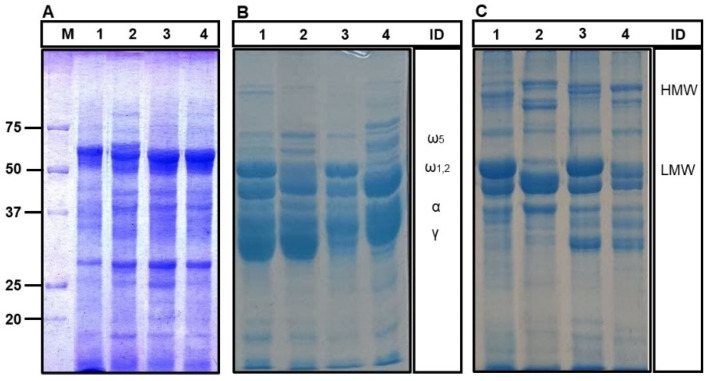
Electrophoresis of Osborne fractions: albumins and globulins (Panel **A**); gliadins (Panel **B**) and glutenins (Panel **C**). Panel **A** was stained in Brilliant Blue Coomassie R250; Panel **B**,**C** with G250. M: Molecular markers (Precision plus Protein—Biorad); Lane 1: durum wheat cv Antalis; Lane 2: soft wheat cv Altamira; Lane 3: tritordeum cv Bulel; Lane 4: tritordeum cv Aucan; ID: Identification based on Landolfi et al., 2021 [20]. ω5: omega 5 gliadins; ω1,2: omega 1,2 gliadins; α: alpha gliadins; γ: gamma gliadins; HMW: high molecular weight glutenins; LMW: low molecular weight glutenins.

The protein profile under reducing conditions of the Osborne fractionated proteins is presented in Figure 2. Tritordeum cv Bulel showed an electrophoretic profile of all fractions being less complex compared to both Aucan and durum wheat. The salt soluble protein profile (albumins and globulins) (Figure 2A) of both the tritordeum cvs appeared comparable to that of soft wheat, with a higher number of bands than the durum wheat flour. These differences were more pronounced in the lower-molecular-weight region (Mr < 30 kDa) and may be attributed to proteins encoded by the *H. chilense* inherited genome.

The electrophoretic profile of gliadins can be divided into four zones representing the typical regions of ω-, γ-, β-and α-gliadins (Figure 2B). A greater protein variability was detected in Bulel compared to Aucan and the two wheat flours. The lowest number of bands were identified in the Bulel’s gliadins, particularly in the high molecular mobility region (Mr > 50 kDa) where the ω-gliadins migrate. This profile is consistent with the ELISA data showing Bulel characterized by the lowest concentration of gliadin detectable with the R5 antibody (Figure 1 and Figure 2). Aucan showed a greater complexity in the same region even when compared to the two reference wheat flours.

The electrophoretic profile of glutenin fractions varied across the four cvs (Figure 2C) in terms of the number of electrophoretic bands detected and electrophoresis mobility. The high-molecular-weight glutenin subunits (HMW-GSs) are responsible for the gluten supramolecular structure, providing the cysteines involved in the formation of the disulfide-bonded backbone in gluten network, affecting the rheological properties of dough [21]. The presence of HMW-GS in tritordeum is due to the contribution of *H. chilense* locus “Glu-Hch1” gene expression on the chromosome 1Hch [22]. This locus is homologous of the wheat Glu-1 locus and to the barley Hor-3 locus [23]. The *H. chilense* genome promotes a similar effect on gluten strength as the D genome inherited by the wheat species from *Aegilops tauschii* [24].

The region of the low-molecular weight glutenin subunits (LMW-GSs) in the two tritordeum cvs appeared similar between each other and to the durum wheat with a higher number of bands with faster molecular mobility compared to soft wheat. Unlike the control wheat, both the tritordeum cvs showed the presence of two main bands in the region of Mr < 30 kDa, which likely are expressed by the *H. chilense* inherited genome (mother). 

Once again, the electrophoresis showed substantial differences in terms of the overall protein expression between the two tritordeum cvs under evaluation. Since the two tritordeum cultivars share the same *H. chilense* line as mother, while they differ in the line of *T. turgidum* spp. *durum* used as father, these differences should be attributed primarily to the durum wheat inherited genome (Arcadia S.p.A., personal communication).

### 2.2. Digestomics

The simulated gastroduodenal digestion was performed on model breads prepared using refined flours to a 35% starting hydration (Figure S1, Supplementary Materials). Breads were subjected to simulated digestion within a few hours from cooking to avoid any alteration of the starch that would have impaired (affected) the digestion. The R5 immunoreactivity of the four kinds of bread was measured and reported in Figure 1. In all cases, the R5 gliadin content of the bread samples was lower than the respective flour, although being comparable in terms of order of magnitude. This was somewhat expected, due to both the formation of the gluten network and baking-induced protein modifications, which may have partly impaired the protein extraction.

#### 2.2.1. Quantitative Analysis of the End Products of Digestion

The digestion products of bread were quantitatively evaluated. The reducing sugar release (RSR) over duodenal digestion was measured by the enzymatic-spectrophotometric method (Figure S2). The RSR curves of the four bread samples were comparable (*p* > 0.05). Total starch content in tritordeum is knowingly higher than barley and lower than wheat, with a content in resistant starch similar to barley [25]. In the stomach and in the intestine, resistant starch, together with the higher viscosity (due to the presence of soluble fibers), makes the chyme of the barley bread less accessible to the enzymes, reducing the glycemic index compared with the reference wheat bread [6]. The content of β-glucans in tritordeum was found to be five times lower than barley [13]. The quantification of reducing sugars at the end of digestion has only been a side part of this study. We are planning a forthcoming investigation aimed at quantifying the end products of starch digestion to confirm a different behavior for tritordeum bread than from its wheat counterpart. 

Free amino acids, di- and tripeptides are the products of protein digestion that can be transported across the intestinal barrier. The starting content of free amino acids released during bread preparation was measured in the undigested cooked breads (Figure S3). Bread made with the two tritordeum cvs and the durum wheat cv had a starting content of α-amino nitrogen of 0.35% (*w*/*w*), the soft bread was 0.25% and the protein-free bread was 0.15%. The commercial protein-free bread, used as background reference to quantify the endogenous amino acids products of natural gastroduodenal enzymes turnover, had an α-amino nitrogen content < 0.1%, in line with what was declared on the food package label. 

**Figure 3:**
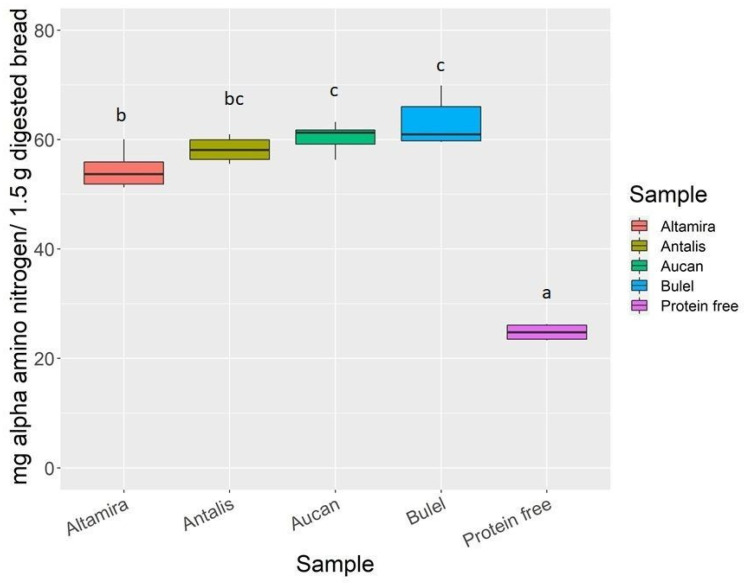
Alpha-amino nitrogen released at the end of the duodenal simulated digestion of 1.5 g of bread obtained from the different cereals. Bars with different letters are significantly different (*p* < 0.05) and the Tukey-test.

The level of α-amino nitrogen determined in the digested protein-free bread accounted for half of the content of the analyzed bread samples on average. This background level is most likely due to the autoproteolysis of the digestive enzymes. This underlines the importance of having protein-free reference matrices to be used as background reference samples. The breads baked with the soft and the durum wheat showed a comparable digestibility (Figure 3). The two tritordeum cvs showed the highest release (*p* > 0.05) of α-amino nitrogen related to the protein concentration of the flour determined by Kjeldahl. 

#### 2.2.2. Qualitative Evaluation of the Peptides Resistant to Digestion

The availability of well annotated and curated protein sequence databases is essential for inferring relevant information from mass spectrometry data. The analysis of cereal seed storage proteins is challenging because of the natural polymorphism, with a high number of protein isoforms differing by point mutations, and the homology across cvs and species [26]. Tritordeum, being a novel crop, lacks a protein database. Therefore, the identification of the proteins was performed using a combined database of the two parent proteomes, *H. chilense* and *Triticum turgidum* spp. *durum*, and of *H. vulgare*.

The tritordeum bread derived peptides, resistant to gastroduodenal digestion primarily belonging to α-amylase inhibitors (AAI) and to the glutenin family (Table 2, Tables S1 and S3). The AAI are knowingly resistant to gastroduodenal digestion, mainly due to the presence of disulphide bridges that stabilize the polypeptide chain [27,28], and are involved in IgE-mediated wheat (Tri a 28–39) and barley food allergies (Hor v 15) [29,30]. 

Several peptides were identified as derived from *Triticum* proteins, and fewer were associated with the *Hordeum* (Tables S1 and S3). Peptides belonging to the γ-3-ordeins (Uniprot ID: Q6EEY5) and the D-hordein (Uniprot ID: B0L965) from *H. chilense* could be identified in both digests of tritordeum bread. Unique proteins to tritordeum cv Aucan and Bulel were identified. AAI and glutenin subunits may be suggested as suited species markers for discriminating between the Bulel and the Aucan varieties. As expected, the unique proteins were expressed from the *Triticum* father line differing between the two tritordeum. Interestingly, despite the common *Hordeum* mother, a gamma prolamin of the *Hordeum brachyantherum* subsp. *brachyantherum* (mother) could be uniquely identified by homology in cv Bulel.

**Figure 4:**
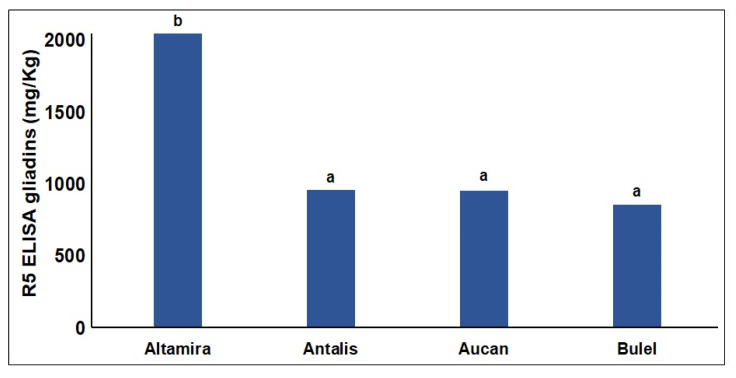
Quantification of the “QQPFP” celiac toxic motif recognized by the R5 monoclonal antibody in duodenal digests (mg of gliadins per kg of the soluble duodenal digest) of bread obtained from different cereals. Bars with different letters are significantly different (*p* < 0.05) and the REGW-F test.

The immunoreactivity of duodenal digests determined using a competitive R5-competitive ELISA was 50% lower in digested tritordeum and durum wheat bread samples compared with the soft wheat bread (*p* < 0.05) (Figure 4).

While the R5 immunoreactivity of Aucan flour and bread was comparable with the soft wheat flour, the digests of tritordeum cv Aucan bread had an immunoreactivity comparable with that of tritordeum cv Bulel and the durum wheat digests. The analyses were performed on the soluble digest, which is likely the fraction to be uptaken in the gut. The lower R5-immunoreactivity of Bulel digest is in line with previous literature data [20]. The reduced immunoreactivity of the Aucan bread duodenal digest may be explained by a low digestion level that could have spared large protein fragments carrying the R5-epitope(s) trapped in the insoluble fraction. This fraction is not uptaken by enterocytes and represents the primary fermentation substrate of gut microbiota. A recent in-vivo study, showed a significant decrease of gluten intestinal peptides, determined by ELISA, in the stool of subjects fed with tritordeum bread compared with wheat bread-fed subjects [8]. The bread produced with Bulel flour, under our analytical conditions, showed similar results as the in vivo study presented by Vaquero et al., 2018 [8]. The Aucan bread instead behaved in a completely different way suggesting future in vivo studies may need to be designed to include different tritordeum cvs to confirm their suitability for subjects affected by non-celiac gluten sensitivity, especially in consideration of the relative stability of AAI.

Due to the complexity of the mass spectrometry data an in-silico evaluation was carried out only on those peptides identified in both technical replicates, to enhance confidence. Overall, 93 peptides resistant to gastroduodenal digestion identified by mass spectrometry were common to the digests of Aucan and Bulel bread (Tables S3 and S4); 38 and 59 peptides were uniquely identified in the Bulel and Aucan bread digests, respectively (Table S5). Many of the unique peptides were inferred to the unique proteins previously listed in Table 1. Interestingly, one HMW-GS protein (Uniprot accession K4N1X7) was common to the two tritordeum cvs. The majority of the peptides identified in both digests mapped to the same protein regions, however, they had different N- and C-terminal trimming, therefore were assigned as unique (Figure S5). Two peptides with sequences, 130-QSGQGQQPGQGQQP-143 and 213-QSGQGQQPGQGQPG-226 were uniquely identified in Aucan and were located in the N-terminal region of the protein. No peptides were identified in the same protein region among the Bulel-derived peptides. In contrast, the peptide with sequence 342-SLQQPGQGQQPGQGQPG-358 was identified only in Bulel. These misidentifications may be due to the bioinformatic protein inferring process, that would only list peptides with 100% identity. Wheat proteins are characterized by high polymorphism and the presence of several protein sequences differing by few amino acids [26]. The protein assignment informs about the presence of a protein family rather than a specific protein, especially for gluten proteins. In this case, it may indicate the presence of two isoforms of the HMW-GS (K4N1X7) expressed in the two tritordeum cvs, carrying mutations in the two identified regions. Two studies previously attempted to map the products of tritordeum cvs that had undergone simulated digestion, working either on isolated proteins [8] or the flour [20]. This is the first study mapping the digestion products of model bread prepared with tritordeum flour, using the INFOGEST standardized model [17]. 

**Figure 5:**
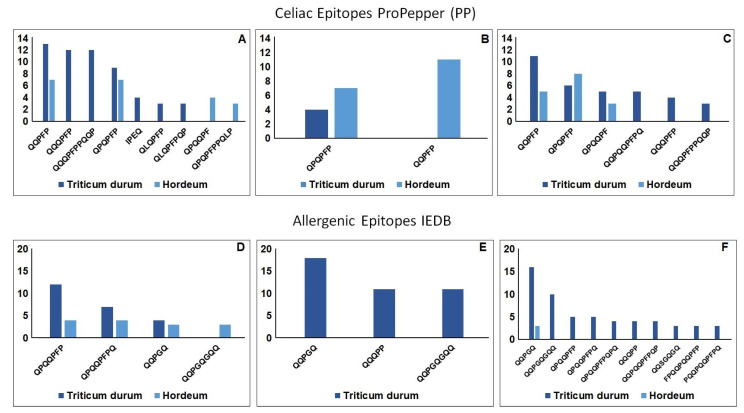
Graphical representation of the peptides surviving the digestion with potential adverse effects on human health. (**A**) number of CD epitopes (ProPepper) common epitopes found in digests of bread baked with the two tritordeum cvs); (**B**) number of CD epitopes (ProPepper) found uniquely in digests of tritordeum cv Bulel bread; (**C**) number of CD epitopes (ProPepper) found uniquely in digests of tritordeum cv Aucan bread; (**D**) number of allergenic epitopes (IEDB) common epitopes found in digests of bread baked with the two tritordeum cvs; (**E**) number of allergenic epitopes (IEDB) found uniquely in digests of tritordeum cv Bulel bread; (**F**) number of allergenic epitopes (IEDB) found uniquely in digests of tritordeum cv Aucan bread. Only epitopes identified in at least 3 precursor peptides were reported.

The in-silico epitope analysis showed a larger number of peptide precursors of celiac toxic motifs and IgE binding peptides for the Aucan bread than for the Bulel counterpart (Figure 5). The analysis of epitopes also showed the prevalent contribution of *Triticum* in the overall allergenicity/celiacogenic potential of tritordeum bread.

The sequence analysis (Figure 5 and Figure S4) of digestion-resistant peptides showed the high frequency among others of QQPFP, QQPYP, PQPFP sequences, which are targets of the R5 competitive ELISA. The mapping of the R5-epitopes within the protein sequences from the two tritordeum cvs highlighted the prevalent contribution of *Triticum*-derived sequences in Aucan and *Hordeum*-derived sequences in Bulel.

## 3. Materials and Methods

### 3.1. Grains and Flours Production

A field study was carried out on the north-west Italian plain at Cigliano (45°18′ N, 08°1′ E; elevation 237 m), in the 2019–2020 growing season. The experiment was performed on a silty-loam soil sub acid, characterized by a medium cation-exchange capacity and organic matter content. In the same experimental field, the following genotypes have been cultivated side by side:

- A soft wheat (hexaploid AABBDD), cv named Altamira (seeds provided by Limagrain Italia S.p.A., Busseto, Italy) classified as ordinary bread-making wheat [31] registered in the Italian varietal list in 2009 (https://www.sian.it/mivmPubb/listeVarieta.do; Sian code: 11239; consulted on the 20 December 2021) and widely cultivated in Italy;
- A durum wheat (tetraploid AABB), cv named Antalis (seeds provided by CGS Sementi S.p.A., Acquasparta, Italy), characterized by medium-high GPC and gluten index; registered in the Italian varietal list in 2014 and widely cultivated in Italy;
- Tritordeum (hexaploid AABBHchHch), cv named Bulel (seeds provided by Arcadia S.p.A., Pamplona, Spain), which was registered in the CPVO (Community Plant Variety Office) List in 2015;
- Tritordeum (hexaploid AABBHchHch), cv named Aucan (seeds provided by Arcadia S.p.A., Pamplona, Spain), which was registered in the CPVO List in 2013.

The treatments were assigned to experimental units using a completely randomized block design with four replicates. The plot size was 7 × 1.5 m. 

The same agronomic techniques have been adopted for all cvs. Briefly, the previous crop was maize and planting was performed in 12 cm wide rows at a seeding rate of 400 seeds m^−2^ on November 6th 2019, following an autumn plowing (30 cm) and disk harrowing to prepare a proper seedbed. A N fertilization treatment of 130 kg N ha^−1^ was used on all the cultivated samples. The total N rate for each treatment was top-dressed applied as a granular ammonium nitrate fertilizer, split 50 kg N ha^−1^ at tillering (growth stage, GS23) and 80 kg N ha^−1^ at the beginning of stem elongation (GS32). The foliar diseases were controlled by applying a fungicide (pyraclostrobin 150 g ha^−1^ and fluxapyroxad 75 g ha^−1^, Priaxor^®^, BASF Agricultural Solutions) at booting stage (GS45). Harvesting was carried out with a Walter Wintersteiger cereal plot combine-harvester on 29 June 2020. 

Grains (2 kg) from each plot and cv were milled using the Bona 4RB mill (Bona, Monza, Italy) to obtain refined flour, (tritordeum and soft wheat) and semolina (durum wheat). GPC (Kjeldahl N × 5.7, on a dry matter basis) and ash content were determined according to Blandino et al., 2015 [32] on grains collected at the commercial maturity stage. Grains (2 kg) from each plot and cv were milled using the Bona 4RB mill (Bona, Monza, Italy) to obtain refined flour. 

### 3.2. Materials

All the reagents used in this study were of analytical or higher grade. Sodium phosphate, ammonium bicarbonate (AmBic), acetic acid and the other chemicals used to produce the simulated salivary fluid (SSF), the simulated gastric fluid (SGF) and the simulated intestinal fluid (SIF), were provided by Carlo Erba (Milan, Italy). The enzymes used for in vitro human digestion were purchased from Sigma (St Louis, MO, USA), in line with those recommended by the INFOGEST protocol [33]. Trichloroacetic acid (TCA), sodium dodecyl sulfate (SDS), glycerol, tris(hydroxymethyl) aminomethane hydrochloride (Tris-HCl), ethylenediaminetetraacetic acid (EDTA), guanidine chloride, trifluoroacetic acid (TFA), formic acid (FA), acetonitrile (ACN), 2-vinylpyridine monomer, and *p*-toluenesulfonyl-l-arginine methyl ester (TAME) were also from Sigma-Aldrich. Egg lecithin was purchased from Lipid Products (Redhill, UK). The electrophoresis reagents were all obtained from Bio-Rad (Milan, Italy).

The protein-free bread (<0.1%, *w*/*w*) (Amino’ pane le rosette produced by Antica Farmacia Orlandi) was purchased from a local pharmacy.

### 3.3. Quantification of Protein in Flour and Semolina

The Kjeldahl analysis was performed as described by Abrams et al., 2014 [34], with some modifications. Two grams of each flour were weighed in a Kjeldahl tube, in which a mixture of copper and potassium sulphate (0.5 g and 12 g, respectively) and 20 mL of 96% sulfuric acid were added. The mineralization was performed following a thermal ramp: 230 °C for 20 min, 290 °C for 45 min, 320 °C for 35 min, and 420 °C for 60 min. The sample was diluted with 50 mL of deionized water, and 90 mL of 45% NaOH were added. The solution of the ammonia was distilled over steam and collected in a flask containing 50 mL of 4% boric acid. The total nitrogen was determined by titration with 0.1 N HCl, after adding a mixed indicator (methyl red 0.1% and bromocresol green 0.2% in ethanol). A conversion factor of 5.7 was used to convert the total nitrogen to total protein and the results were expressed as g of total protein over 100 g of sample. Samples were analyzed in biological duplicates.

### 3.4. Gliadin Quantification with R5 Commercial ELISA

Flour and bread samples were analyzed with the RIDASCREEN^®^ Gliadin (Art. No. R7001, R-BIOPHARM AG, Darmstadt, Germany), which is a sandwich enzyme immunoassay (ELISA) based on R5 monoclonal antibody recognising the “QQPFP” celiac toxic motif. Proteins were extracted in the Cocktail (patented) recommended by Codex Alimentarius for the optimized extraction of gliadin from heat-processed and non-heated food samples (Art. No.: R7006/R7016, patent WO 02/092633, R-BIOPHARM AG, Darmstadt, Germany), which was used according to the manufacturer’s instructions, and according to the AOAC Official Method of Analysis for gluten detection (OMA 2012.01).

RIDASCREEN^®^ Gliadin competitive (Art. No. R7021, R-BIOPHARM AG, Darmstadt, Germany) was used to analyse the products of in vitro bread digestion, according to the manufacturer’s instructions. 

The gastroduodenal digestion products were analyzed in duplicate.

### 3.5. Osborne Fractionation

The Osborne fractionation was performed as previously described in Landolfi et al. 2021 [20]. The albumins and globulins were solubilized from the non-defatted flour (1:10, *w*/*v*) in 100 mM KCl, 50 mM Tris-HCl pH 7.8, and 5 mM EDTA for 4 h at room temperature (−20 °C). The solution was centrifugated for 15 min at 3500× *g* and the supernatants from two consecutive extractions were pooled. The gliadins were extracted 1:10 *w*/*v* with 70% (*v*/*v*) ethanol for twelve hours at room temperature (−20 °C). Glutenin extraction was performed at 60 °C for 30 min, in 50% *v*/*v* 1-PrOH + 50 mM Tris-HCl (pH 8.5) + 1% (*w*/*v*) 1,4-Dithio-d-threitol (DTT). The cysteine residues of the glutenin extracts were pyridyl-ethylated at 60 °C for 15 min with 2-vinylpyridine.

### 3.6. 1-Dimensional Electrophoresis (SDS-PAGE)

Purified protein fractions were separated by SDS-PAGE under reducing conditions, using a Mini-PROTEAN cell systems (Bio-Rad). To this purpose, proteins were precipitated in cold (−20 °C) propan-2-one (1:4, *v*/*v*), suspended in the SDS-PAGE Laemmli Buffer (0.125 M Tris–HCl pH 6.8, 5% SDS, 20% glycerol, 5% (*w*/*v*) 2-sulfanylethanol, 0.02% bromophenol Blue) and boiled in a water bath for 5 min. After quantification with a micro-Lowry kit (Sigma-Aldrich, Saint Loius, MO, USA), 25 µg of gliadins, 75 µg of glutenins, and 75 µg of albumins/globulins were loaded onto a 12% acrylamide gel (Bio-Rad). Migration of proteins was conducted at 120 V for 10 min and 220 V for 35 min. Afterwards, gels were fixed with TCA (24%) overnight (16 h) and stained with Coomassie^®^ Brilliant Blue R-250 (gliadins and glutenins) and G-250 (albumins and globulins).

### 3.7. Preparation of the Model Breads

Model breads were prepared using commercial baker’s yeast (*Saccharomyces cerevisiae*). The same recipe was used for all the flours from different cvs. Flour (50 g) was mixed with 33 g of water and 5 g of yeast and 7 g of salt were added. The mixture was allowed to stand at 20 °C for 12 h. Loaves were baked at 230 °C for 40 min. After cooling, loaves were cut into slices and subjected to in vitro digestion within a few hours to avoid any alteration of the digestibility due to storage conditions (e.g., starch retrogradation due to freezing) [35]. 

### 3.8. Static Oral-Gastric-Duodenal Digestion of Model Breads

In vitro oral and gastroduodenal digestion was carried out using the harmonized and standardized INFOGEST method [33]. The trypsin activity of the porcine pancreatin was determined using the *p*-toluene-sulfonyl-l-arginine methyl ester (TAME) as the substrate according to Brodkorb [17] and measuring the absorbance at 247 nm for 10 min. The trypsin activity was found to be 9.5 U/mg of powder. At the end of the duodenal digestion, samples were boiled for 5 min to interrupt the enzymatic digestion and centrifuged at 7900× *g*, for 30 min. The supernatant containing digestion products, which are likely to be absorbed by enterocytes, was collected, and processed for further analysis, including peptidomics and α-amino nitrogen determination. Aliquots of duodenal digests were collected every 30 min.

### 3.9. Preparation of Samples for the Alpha Amino Nitrogen Determination

The solubilization of proteins from the cooked bread samples and protein-free bread was performed in the SSF without enzymes (1:12, *w*/*v*) for 3 h at 37 °C. Prior to α-amino nitrogen determination, all sample were de-proteinized TCA up to a final concentration of 20% (*w*/*v*). After the protein precipitation was conducted for 30 min at 20 °C, the solution was centrifuged at 4000× *g* for 30 min, 4 °C and neutralised to a pH 7 with 1 N NaOH prior to analysis.

The content of free α-amino nitrogen in the samples was determined using the EnzytecTM Alpha-amino Nitrogen kit by R-Biopharm (E2500 R-Biopharm AG, 64297 Darmstadt, Germany) following the manufacturer’s instructions. The iCubio i-Magic M9 (Origlia S.r.L, 20007 Cornaredo, Italy) was set to perform the enzymatic reaction in full automatization and the absorbance was read at 340 nm. All samples were assayed in triplicate and absorbance values were averaged.

### 3.10. Free Glucose Quantitative Determination

D-glucose was quantified directly in the soluble digest using the Enzytec™ Liquid D-Glucose kit by R-Biopharm (E8140 R-Biopharm AG, 64297 Darmstadt, Germany), following the manufacturer’s instructions. The analyses were performed on the iCubio i-Magic M9 (Origlia S.r.L, 20007 Cornaredo, Italy) as described for the α-amino nitrogen (Section 3.9). All samples were assayed in triplicate and absorbance values were averaged.

### 3.11. Preparation of Peptides for Mass Spectrometry Analysis

Peptide digests were desalted using C18 Sep-Pak 360 mg sorbent weight (WAT051910, particle size 55–105 µm, pore size 125 Å) (Waters Co., Milford, MA, USA). The equilibration and cleaning phases were carried with a 0.1% TFA in water. Peptides were eluted with 70% acetonitrile (*v*/*v*) containing 0.1% TFA (*v*/*v*).

### 3.12. Liquid Chromatography-Tandem Mass Spectrometry (LC/MSMS) Analysis 

LC-MS/MS analysis was performed by using a Q Exactive Orbitrap mass spectrometer (Thermo Scientific, San Jose, CA, USA), online coupled with Ultimate 3000 ultra-high performance liquid chromatography equipment (Thermo Scientific, 95134 San Jose, CA, USA)). Samples were loaded through a 5mm long 300 µm id pre-column (LC Packings, San Jose, CA, USA) and separated by an EASYSpray™ PepMap C18 column (2 µm, 25 cm × 75 µm) 3 µm particles, 100 Å pore size (Thermo Scientific, San Jose, CA, USA). Eluent A was 0.1% formic acid (FA) (*v*/*v*) in water; eluent B was 0.1% FA (*v*/*v*) in 80% (*v*/*v*) ACN. The column was equilibrated at 5% B. Peptides were separated applying a 5–40% gradient of B over 60 min. The flow rate was 300 nL/min. The mass spectrometer operated in data-dependent mode and all MS1 spectra were acquired in the positive ionization mode with an *m*/*z* scan range of 350 to 1600. Up to 10 of the most intense ions in MS1 were selected for fragmentation in MS/MS mode. A resolving power of 70,000 full width at half maximum (FWHM), an automatic gain control (AGC) target of 1 × 10^6^ ions and a maximum ion injection time (IT) of 120 ms were set to generate precursor spectra. MS/MS fragmentation spectra were obtained at a resolving power of 17,500 FWHM. To prevent repeated fragmentation of the most abundant ions, a dynamic exclusion of 10s was applied. Ions with one or over six charges were excluded. 

A specific database was generated for the analysis of the MS/MS data. The database included UniprotKB entries for *Triticum turgidum* spp. *durum*, *Hordeum chilense* and *Hordeum spontaneum*, downloaded on the 01/06/2021. The DB Toolkit was used to customize the database and remove redundant sequences [36,37]. The *Sus scrofa* protein sequences, downloaded on the 16/10/2019 from UniprotKB, were also included in the database to detect contaminants, thus increasing the confidence of identification of the Tritordeum-derived peptides.

PEAKS Studio (version 6.0, Bioinformatics Solution Inc., 202-140 Columbia St W, Waterloo, ON, Canada) was used for database searching, applying the following parameters: oxidation on methionine, deamidation on the glutamine and asparagine, and pyroglutamic for N-terminus glutamine as variable modifications; mass tolerance value of 8 ppm and 0.02 Da for precursor and MS/MS fragment ions, respectively; no cleavage specificity. The peptide-level false discovery rate (FDR) was set at 0.1%. Proteins with score −10LgP > 20 were accepted.

### 3.13. In Silico Analysis of Peptides Resistant to Digestion

Peptides identified at the end of the gastroduodenal digestion were in silico evaluated for their celiacogenic potential and IgE capacity. IgE binding sequences were retrieved from the free Immune Epitope Database (IEDB) (https://www.iedb.org/, downloaded on the 20 September 2021). 

The celiac toxic motif was retrieved from the ProPepper database (https://www.propepper.net/, downloaded on the 20 September 2021) [38]. These epitopic/celiacogenic sequences were manually searched in the pool of resistant peptides identified by MS in the duodenal digests of Bulel and Aucan breads.

To increase the confidence of identification and the strength of the *in-silico* analysis, the epitopic/celiacogenic sequences were searched, only considering the peptides identified in both technical replicates. The analyses were performed with the peptides common to the tritordeum cvs and those uniquely identified in Bulel and Aucan. Peptides resulting from digestion that belonged to the same protein were aligned with Clustal Omega (https://www.ebi.ac.uk/Tools/msa/clustalo/, accessed on 20 December 2021) and graphically evaluated using the WebLogo software (https://weblogo.berkeley.edu/logo.cgi, accessed on 20 December 2021) to highlight the recurring regions. This analysis was aimed at aligning in silico the surviving peptides to their toxic-allergenic potential. 

### 3.14. Statistical Analysis

Statistical analysis was performed using SPSS statistic v.27 Chicago: SPSS Inc. Data were compared by one-way analysis of variance (ANOVA), followed by the Tukey–Kramer post hoc test (α = 0.05) for all analysis and Ryan–Eino–Gabriel–Welsch F test (α = 0.05) for the ELISA analysis.

## 4. Conclusions

This study examined for the first time the complex proteome of the tritordeum, highlighting the subtle but technologically and nutritionally relevant differences in the protein set of two commercially mainstream tritordeum cvs, namely Aucan and Bulel. The inter-cvs differences observed may be attributable to the different contributions of the *Triticum turgidum* spp. *durum* genome. Our results suggest that attention should be paid in considering all the tritordeum cvs as a *unicum* in terms of protein expression, since in some cases the protein contribution can vary along the genomic characteristics of the *Hordeum* and *Triticum* parents. 

In the same way, the first in vitro digestomic analysis carried out on bread baked with tritordeum flour in the present study evidenced that the process of digestion produced different peptidomes, with possible different outcomes in terms of immunoreactivity and allergenicity.

## Figures and Tables

**Table 1:** Ash and grain protein content (GPC) and total protein content of the flour (TPC).

Species	Cv	Ashes (%)	GPC (%)	TPC (%)
Soft wheat	Altamira	1.89 ± 0.04 ^b^	11.17 ± 0.23 ^a^	8.65 ± 0.01 ^a^
Durum wheat	Antalis	1.82 ± 0.02 ^a^	11.66 ± 0.35 ^b^	9.19 ± 0.21 ^ab^
Tritordeum	Aucan	1.96 ± 0.02 ^c^	13.43 ± 0.15 ^c^	10.63 ± 0.33 ^c^
Tritordeum	Bulel	1.86 ± 0.04 ^ab^	11.97 ± 0.07 ^b^	9.84 ± 0.3 ^bc^

Values followed by different letters are significantly different (*p* < 0.05).

**Table 2:** LCMSMS identified proteins in tritordeum bread digests. Only proteins identified in both technical replicates were taken into consideration to increase the confidence in identification. Isoforms were removed and the extensive list of identified proteins is available as Supplementary Materials Tables S1 and S3.

	Accession	Species	−10LgP	Coverage (%)	Peptides	Description
**PROTEINS IDENTIFIED IN BOTH TRITORDEUM DIGESTS**	Q9XGF0	TRITD	74.72	20	12	LMW-GS
	A0A446W0B5	TRITD	72.20	14	7	AAI
	K4N1X7	TRITD	74.67	10	8	HMW-GS
	A0A446W0A1	TRITD	76.67	12	9	AAI
	H8Y0D1	TRITD	68.82	15	9	Alpha prolamin
	A0A446W0B4	TRITD	63.66	12	4	UNP
	A0A446W085	TRITD	71.15	11	7	AAI
	A0A446TL77	TRITD	39.57	5	2	rRNA N-glycosidase
	A0A446W0C7	TRITD	51.01	9	3	AAI
	A0A446V2J2	TRITD	42.65	4	2	AAI
	A0A446V2Q9	TRITD	45.34	8	3	AAI
	Q6EEY5	HORCH	40.78	8	3	Gamma 3 hordein
	B0L965	HORCH	31.52	2	1	D-hordein
	A0A446YMF0/M0WF36	TRITD/HORVV	21.54	4	1	UNP
	A0A287EEX5	TRITD	40.07	6	3	UNP
						
**PROTEIN IDENTIFIED ONLY IN TRITORDEUM CV BULEL**	A0A446JGR8	TRITD	63.12	8	5	AAI
	A0A0E4G9A4	TRITD	48.57	6	5	HMW-GS
	H8Y0M9	HORBR	37.19	12	3	Gamma prolamin
	A0A7H1K1W3	TRITD	31.27	7	2	AAI
	A0A446IHD3	TRITD	20.67	6	1	AAI
	A0A446IHC0	TRITD	31.50	4	1	AAI
						
**PROTEINS IDENTIFIED IN TRITORDEUM CV AUCAN**	A0A2L1K3K6	TRITD	77.43	12	11	HMW-GS
	Q41603	TRITD	44.31	9	3	LMW-GS

TRITD = *Triticum turgidum* subsp. *durum*; HORCH = *Hordeum chilense*; HORBR = *Hordeum brachyantherum* subsp. *brachyantherum*; HORVV = *Hordeum vulgare*; UNP = uncharacterized protein; LMW-GSs = low molecular weight-glutenin subunits; HMW-GSs = high molecular weight-glutenin subunits; AAI = α-amylase inhibitors.

## Data Availability

Mass spectrometry raw data are available from the corresponding author upon request. The identifications and elaborations are provided as Supplementary Supporting Materials.

## Supplementary Materials

The following are available online, Figure S1: (A) Breads prepared with 100% reference flour; (B) Sliced bread. Tritordeum bread appeared yellower than soft wheat. Tritordeum Baked breads showed a comparable alveolation to soft wheat bread, cv Altamira. Figure S2: Reducing sugar release (expressed as mg of glucose) from 1.5 g of digested breads. Error bars represent the variability over two breads digested in two days and two technical replicates. (Panel A) kinetic of breads duodenal digestion (0–30–60–90 and 120 min); (Panel B) mg of reducing sugar released after 4 h of gastroduodenal digestion of 1.5 g of bread. Figure S3: mg of alpha amino nitrogen determined in 1.5 g of the cooked breads. Bars with different letters are significantly different (*p*-value < 0.05) and the REGW-F test. Figure S4: Graphical representation of the peptides surviving digestion. Peptides belonging to the same protein region were aligned and the height of the amino acid reflects its abundance in the sequences. The sequence R5-QQPFP sequence was highly repeated in Aucan bread *Triticum* derived peptides, while in Bulel was found highly repeated in *Hordeum*-derived peptides. Sequence logo of unique peptides from Aucan (Panel A,C) and Bulel (Panel B,D) bread duodenal digestomes. The frequency of the sequences is expressed in bits [39]. Figure S5: Coverage of the *Triticum tugidum* spp. *durum* HMW GS (K4N1X7) by digestion-resistant peptides from Aucan bread (Panel A) and Bulel bread (Panel B). The alignment highlights the uniqueness in several cases is due to the hydrolysis of peptides differing by few amino acids (Supplementary Materials Table S5). Table S1: Nano-LC MS/MS identification of the proteins resistant to the in vitro gastroduodenal digestion of Aucan bread. Table S2: Nano-LC MS/MS identification of the proteins resistant to the in vitro gastroduodenal digestion of Bulel bread. Table S3: list of LCMS/MS identified peptides resistant to in vitro digestion of Aucan bread. Table S4: list of LCMS/MS identified peptides resistant to in-vitro digestion of Bulel bread. Table S5: list of LCMS/MS identified peptides resistant to digestion uniquely identified in Aucan and Bulel breads. This list of peptides was used for the in silico evaluation.

## Sample Availability

Bread samples are available in frozen form upon request to the corresponding author. The grains and flours of compared cultivars can be requested to prof. Massimo Blandino at the University of Turin.

## Abbreviations

AAI	alpha amylase inhibitors
ACN	acetonitrile
AGC	automatic gain control
AmBic	ammonium bicarbonate
ANOVA	analysis of variance
cv	cultivar
DTT	1,4-Dithio-D-threitol
ELISA	enzyme-linked immunosorbent assay
EDTA	ethylenediaminetetraacetic acid
FA	formic acid
FDR	false discovery rate
GPC	grain protein content
GS	growth stage
Hch	*Hordeum chilense*
HMW-GS	High-molecular-weight glutenin subunit
IEDB	Immune Epitope Database
IT	injection time
LC-MS/MS	liquid chromatograph-mass spectrometer/mass spectrometry
LMW-GS	Low-molecular-weight glutenin subunit
RSR	reducing sugar release
SDS-PAGE	Sodium dodecyl sulfate-polyacrylamide gel electrophoresis
SGF	simulated gastric fluid
SIF	simulated intestinal fluid
SSF	simulated salivary fluid
TAME	p-toluenesulfonyl-L-arginine methyl ester
TCA	trichloroacetic acid
TFA	trifluoroacetic acid
TPC	total protein content
Tris-HCl	tris(hydroxymethyl) aminomethane hydrochloride
