# Supplementary material for: Tritordeum as an Innovative Alternative to Wheat: A Comparative Digestion Study on Bread
Source: Molecules. 2022 Feb 15;27(4):1308. doi: 10.3390/molecules27041308 (PMC8877140; doi:10.3390/molecules27041308)

Supplementary Figure S1

A) Breads prepared with 100% reference flour. B) Sliced bread. Tritordeum bread appear yellower than soft wheat. Tritordeum Baked breads showed a comparable alveolation to soft wheat bread, cv Altamira.

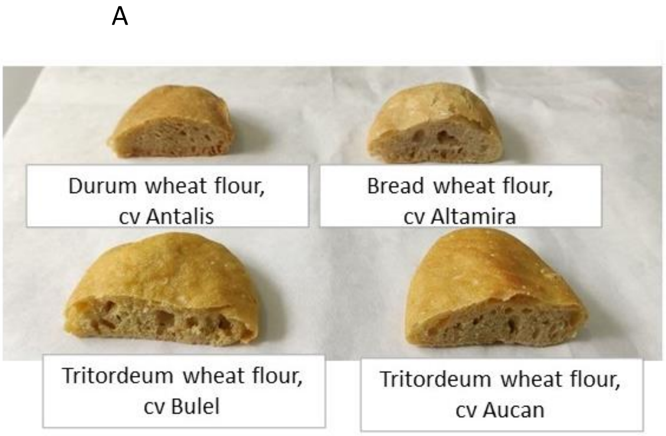

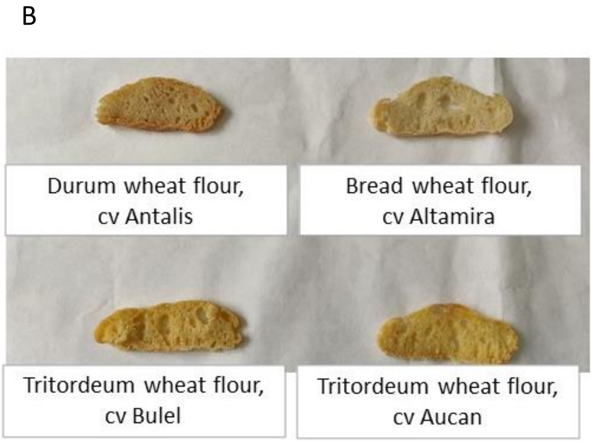

Supplementary Figure S2

Reducing sugar release (expressed as mg of glucose) from 1.5 g of digested breads. Error bars represent the variability over two breads digested in two days and two technical replicates. Panel A) kinetic of breads duodenal digestion (0-30-60-90 and 120 minutes), Panel B) mg of reducing sugar released after 4 hours of gastroduodenal digestion of 1.5 g of bread.

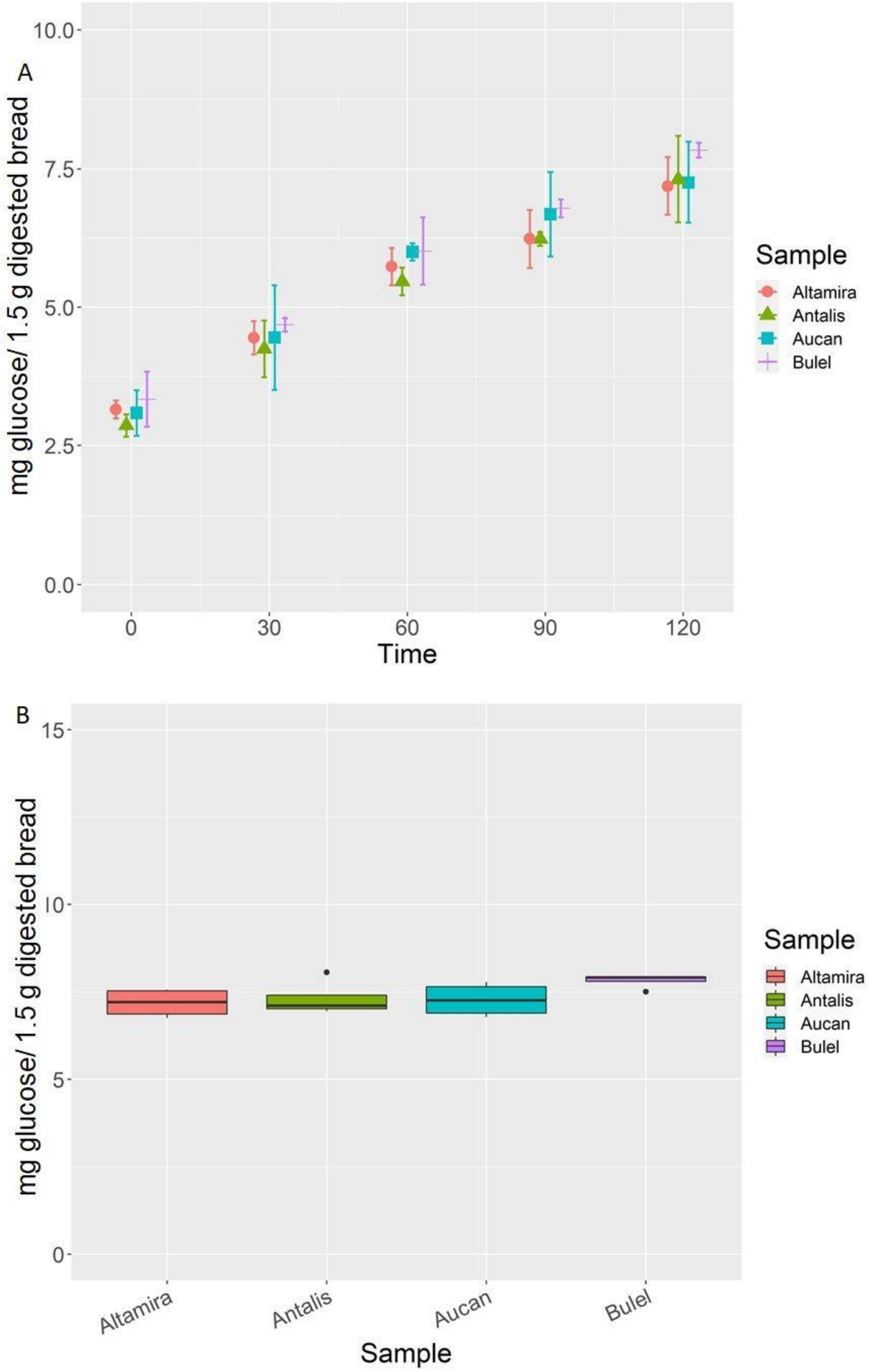

Supplementary figure S3

mg of alpha amino nitrogen determined in 1.5 g of the cooked breads. Bars with different letters are significantly different (p-value < 0.05) and the REGW-F test.

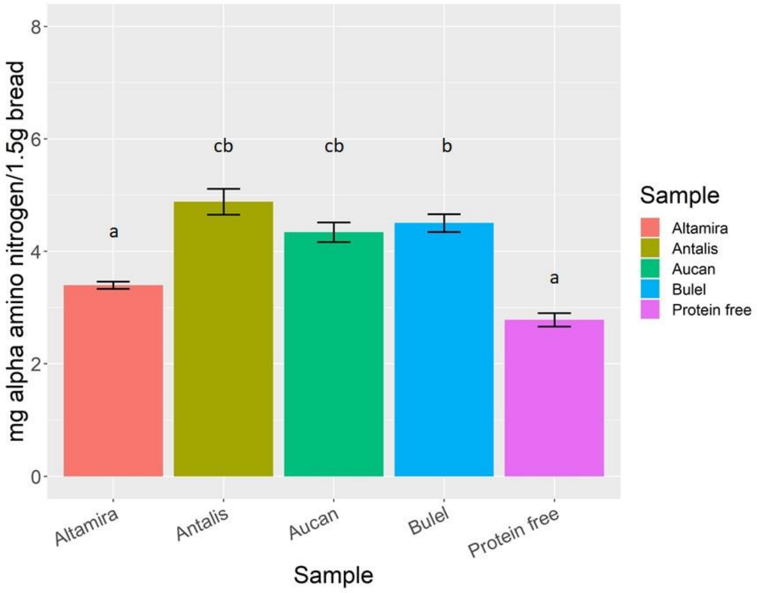

Supplementary figure S4

Graphical representation of the peptides surviving the digestion. Peptides belonging to the same protein region were aligned and the height of the amino acid reflects its abundance in the sequences. The sequence R5-QQPFP sequence was highly repeated in Aucan bread Triticum derived peptides, while in Bules was found highly repeated in Hordeum derived peptides. Sequence logo of unique peptides from Aucan (Panel A, C) and Bulel (Panel B, D) bread duodenal digestomes. The frequency of the sequences is expressed in bits.

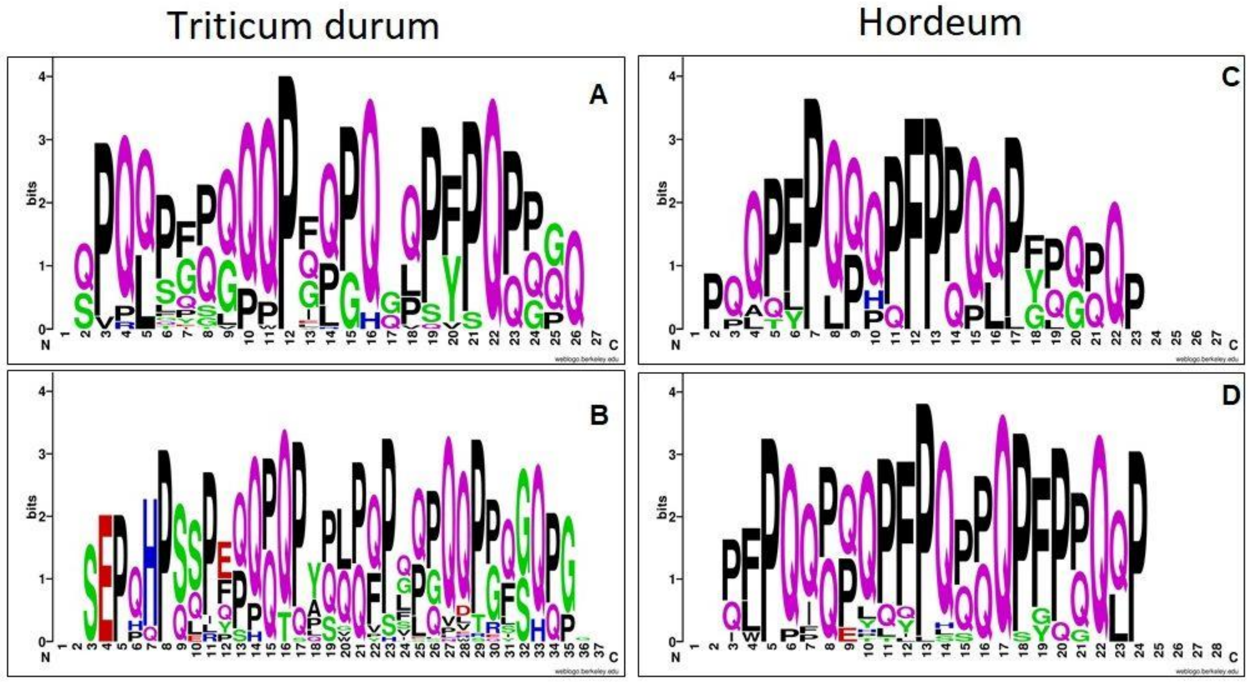

Supplementary figure S5

Coverage of the Triticum durum HMW GS (K4N1X7) by digestion resistant peptides from Aucan bread (panel A) and Bulel bread (panel B). The alignment highlights the uniqueness in several cases is due to the hydrolysis of peptides differing by few amino acids (Supplementary table 5).

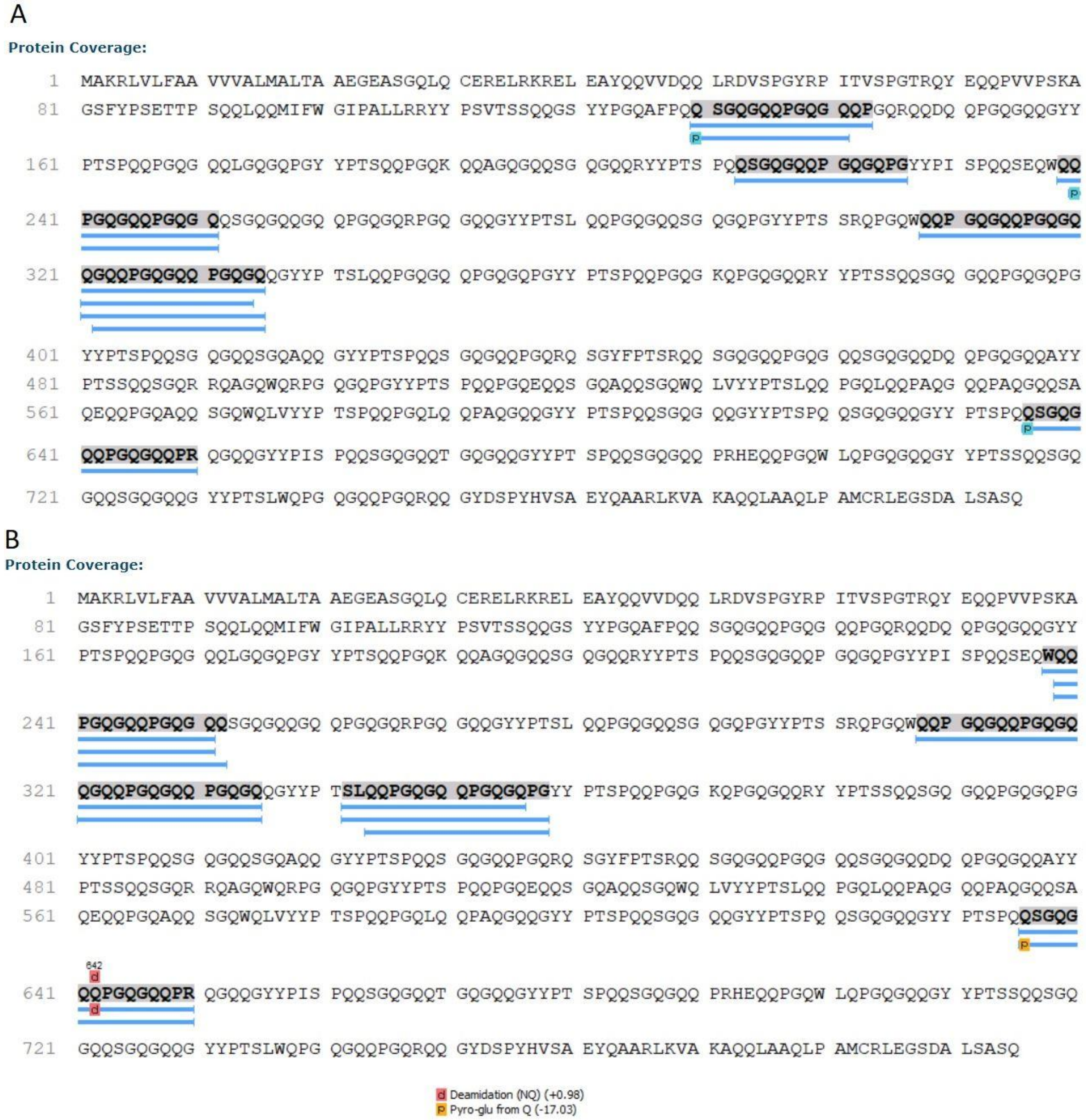

Supplement: Supplementary file 1 [file molecules-27-01308-s001.zip › Supplementary figures for Molecules .pdf]
