# Supplementary figures and images for: Tritordeum as an Innovative Alternative to Wheat: A Comparative Digestion Study on Bread
Source: Molecules. 2022 Feb 15;27(4):1308. doi: 10.3390/molecules27041308 (PMC8877140; doi:10.3390/molecules27041308)

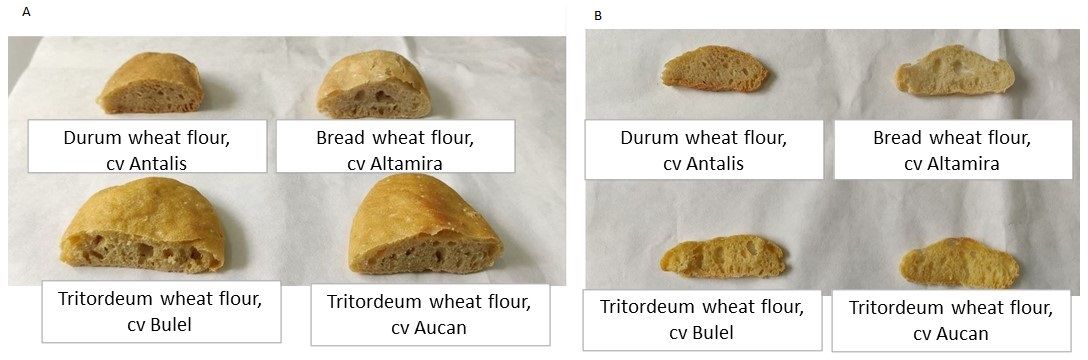

Supplement: Supplementary file 1 [file molecules-27-01308-s001.zip › Supplementary figure S1.jpg]

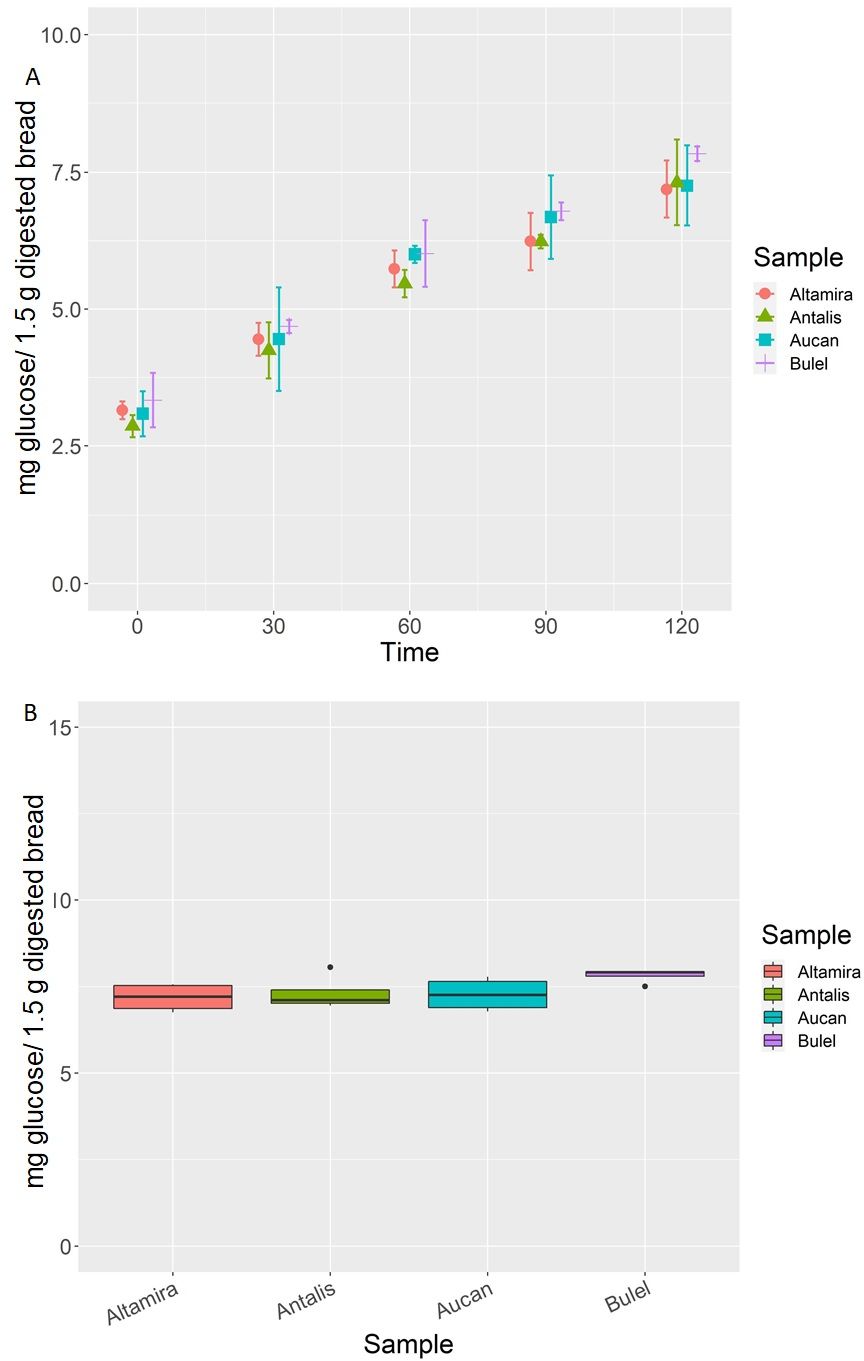

Supplement: Supplementary file 1 [file molecules-27-01308-s001.zip › supplementary figure S2.jpg]

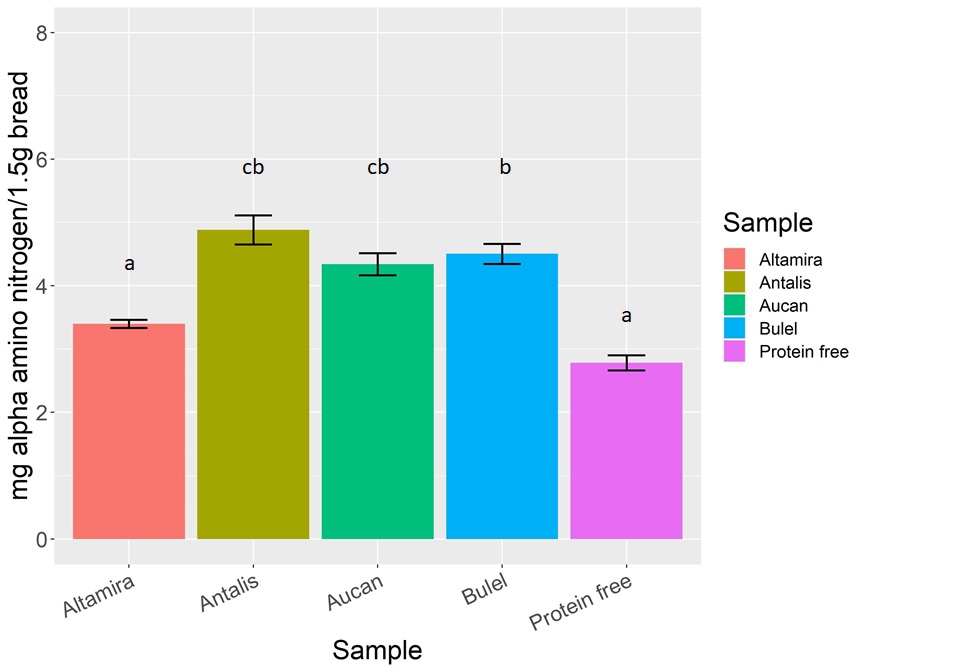

Supplement: Supplementary file 1 [file molecules-27-01308-s001.zip › supplementary figure S3.jpg]

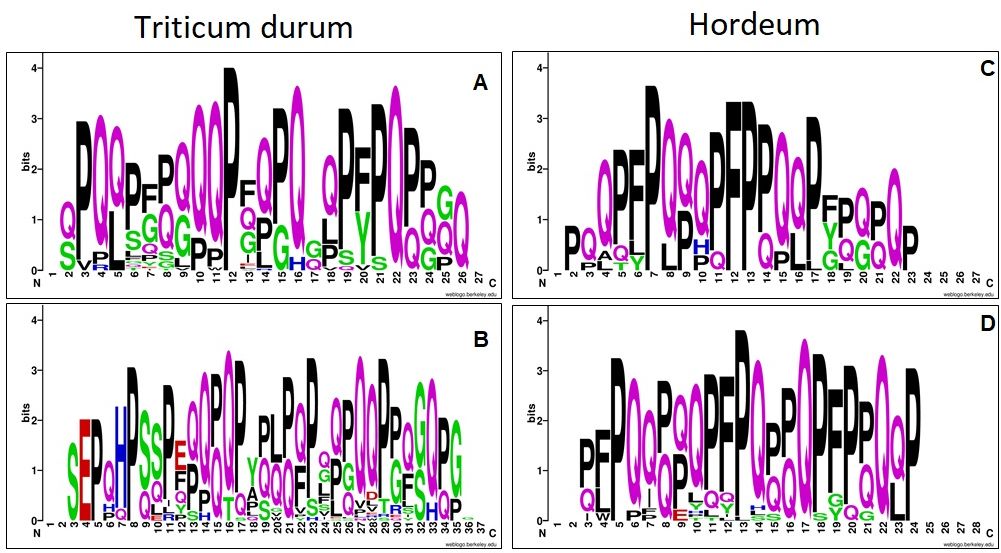

Supplement: Supplementary file 1 [file molecules-27-01308-s001.zip › Supplementary figure S4.jpg]

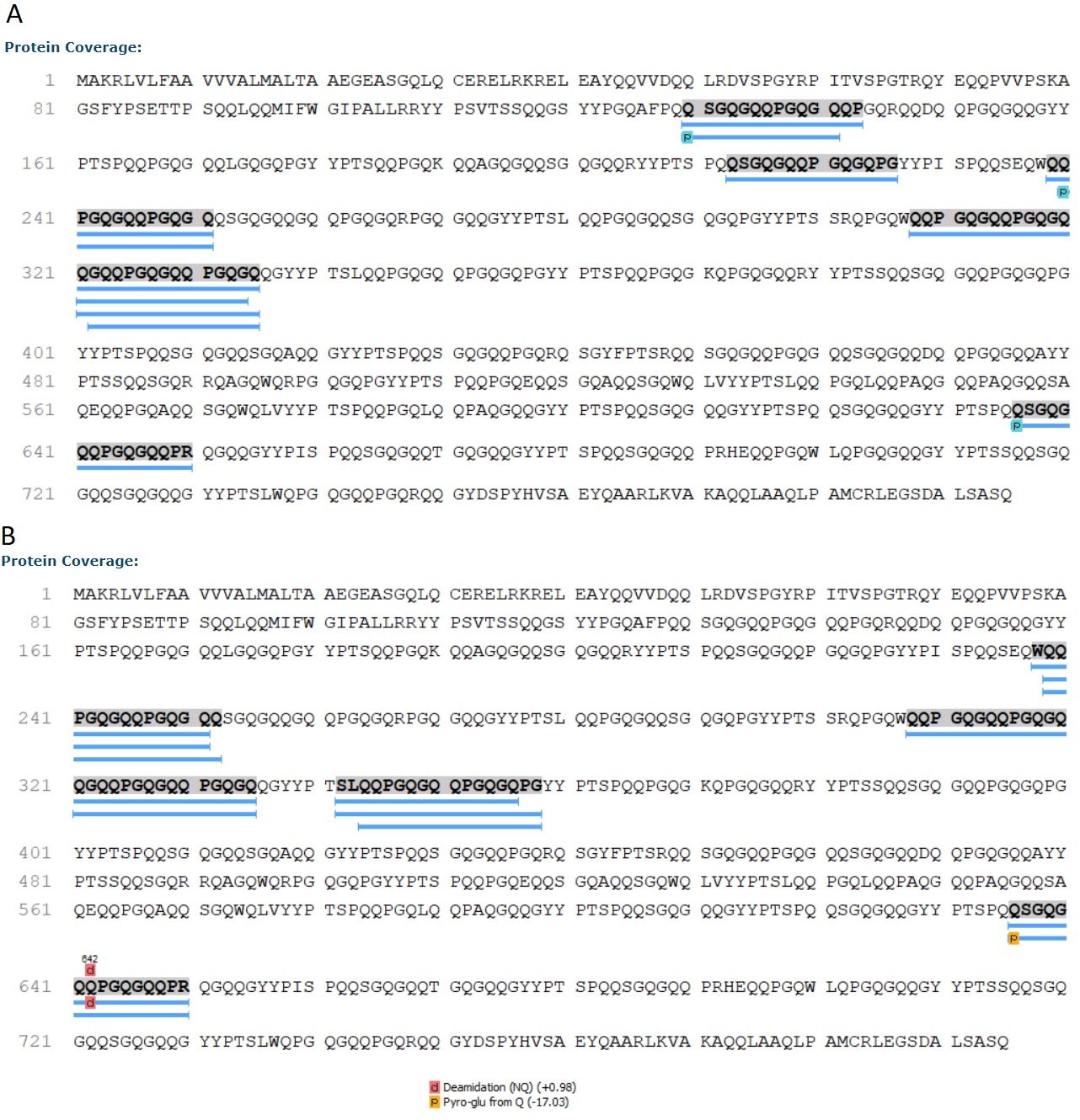

Supplement: Supplementary file 1 [file molecules-27-01308-s001.zip › Supplementary figure S5.jpg]
